# Selective Defect Engineering for Gate-Controlled yet Contact-Transparent Bi_2_O_2_Se Transistors

**DOI:** 10.1021/acsnano.6c04248

**Published:** 2026-05-26

**Authors:** Huynh-Uyen-Phuong Nguyen, Tai-Ting Lee, Yu-Wei Chang, Hung-Chang Hsu, Chih-Yuan Shih, Chi-Chun Cheng, Luc-Phuong-Nhu Tran, Hsin-Chien Chien, Wen-Yuan Fei, Wei-Yen Woon, Yung-Chang Lin, Kazu Suenaga, Yen-Fu Lin, Ya-Ping Chiu, Mei-Yin Chou, Po-Wen Chiu

**Affiliations:** † College of Semiconductor Research, 34881 National Tsing Hua University, Hsinchu 30013, Taiwan; ‡ Department of Electrical Engineering, National Tsing Hua University, Hsinchu 30013, Taiwan; § Department of Physics, 33561 National Taiwan University, Taipei 10617, Taiwan; ∥ 71556 Institute of Atomic and Molecular Sciences, Academia Sinica, Taipei 10617, Taiwan; ⊥ Corporate Research, Research and Development, Taiwan Semiconductor Manufacturing Company (TSMC), Hsinchu 30013, Taiwan; # Nanomaterials Research Institute, National Institute of Advanced Industrial Science and Technology (AIST), Tsukuba 305-8565, Japan; ¶ The Institute of Scientific and Industrial Research (ISIR-SANKEN), Osaka University, Osaka 567-0047, Japan; ∇ Department of Physics, 34916 National Chung Hsing University, Taichung 40227, Taiwan

**Keywords:** Bi_2_O_2_Se transistors, gate-contact trade-off, self-doping suppression, nitrogen-incorporation, contact-transparent

## Abstract

Two-dimensional semiconductors offer a pathway toward ultrascaled electronics, yet achieving strong electrostatic gate control without sacrificing low-resistance contacts remains a fundamental challenge. Here, we report a selective defect-engineering strategy that addresses this gate-contact trade-off in Bi_2_O_2_Se transistors. Low-temperature nitrogen incorporation passivates selenium vacancies through robust N–Bi bonding, suppressing intrinsic self-doping while preserving the intrinsic band dispersion without introducing midgap states. Density functional theory and scanning tunnelling spectroscopy reveal that nitrogen provides acceptor-like compensation by neutralizing vacancy-induced donor states, rather than through conventional substitutional doping. As a result, the Fermi level shifts toward midgap, enabling precise carrier-density modulation while maintaining band-like transport. By spatially confining nitrogen incorporation to the channel region, Bi_2_O_2_Se field-effect transistors are converted from depletion to enhancement mode, achieving high electron mobility and on/off ratios up to 10^9^ while preserving ohmic, contact-transparent injection. This selective defect-engineering approach decouples channel electrostatics from contact properties and provides a potentially scalable, thermally benign route toward gate-controllable, contact-transparent two-dimensional transistors compatible with integrated logic architectures.

## Graphical abstract

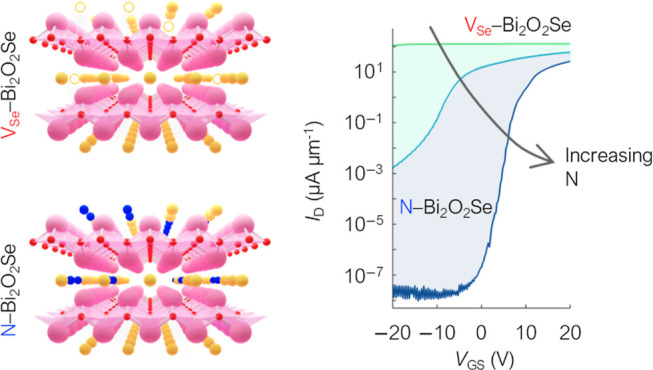

## Introduction

Two-dimensional (2D) semiconductors have emerged as promising building blocks for ultrascaled electronic devices, owing to their atomically thin channels, excellent electrostatic gate control, and compatibility with heterogeneous integration. However, despite rapid progress, a fundamental challenge persists: achieving strong gate modulation without compromising low-resistance and contact-transparent carrier injection. In most 2D semiconductors, reducing carrier density to improve electrostatic control inevitably degrades contact properties, while heavy doping to achieve ohmic contacts leads to degenerate channels with poor gate tunability.^1–19^ This intrinsic gate-contact trade-off has remained a central bottleneck for realizing low-power, logic-compatible 2D transistors. Similar trade-offs between strong gate modulation and efficient carrier injection have been discussed across a range of transistor platforms. Here, examples from organic semiconductors are cited only as conceptual comparisons to illustrate the general nature of the channel-control/contact-transparency trade-off. In the present work, we address this issue in a manufacturable inorganic semiconductor system, demonstrating that strong gate control can be achieved while preserving efficient carrier transport.

Bismuth oxyselenide (Bi_2_O_2_Se) has recently attracted significant attention as a high-mobility 2D semiconductor, combining a silicon-like bandgap, small effective mass (∼0.14 m_0_), and an intrinsic absence of deep defect states.^20–25^ Unlike transition metal dichalcogenides, where chalcogen vacancies introduce midgap traps and Fermi level pinning,^26–28^ selenium vacancies (V_Se_) in Bi_2_O_2_Se donate electrons directly to the conduction band without generating localized in-gap states.^21,29,30^ Recent studies on the Bi–O–Se material family showed that the related layered insulator Bi_2_O_2_Se retains a clean band gap without vacancy-induced surface states and exhibits the thickness-dependent band gap widening characteristic of 2D materials.^44^ This unusual defect chemistry enables band-like transport and low contact resistance but also results in strong self-doping, often rendering Bi_2_O_2_Se channels degenerately conductive and weakly gate-modulated.

Considerable efforts have been devoted to mitigating this self-doping through growth-condition engineering. Chemical vapor deposition (CVD) synthesis using Bi_2_Se_3_ and Bi_2_O_3_ precursors reveals that Se-poor conditions produce degenerately doped films with low contact resistance but poor gate controllability due to abundant V_Se_ donors. In contrast, Se-rich growth suppresses V_Se_ formation and yields more insulating films dominated by bismuth vacancies (V_Bi_), but also introduces intercalated selenium (Se_in_) defects that create midgap states and scatter carriers.^31–35^ As a result, optimizing Bi_2_O_2_Se growth inherently involves a trade-off between gate controllability and transport performance, highlighting the need for a postgrowth strategy that can selectively modulate carrier density.

Here we show that the gate-contact trade-off in Bi_2_O_2_Se can be addressed through a selective defect-engineering strategy based on low-temperature nitrogen incorporation. Rather than globally modifying carrier density during crystal growth, nitrogen is introduced postsynthesis to selectively passivate V_Se_, suppressing intrinsic self-doping while preserving the intrinsic band dispersion without introducing midgap states. Density functional theory (DFT) calculations reveal that nitrogen preferentially chemisorbs at vacancy sites through strong N–Bi bonding, providing acceptor-like compensation via donor-vacancy neutralization. Crucially, this process enables spatially selective modulation of carrier density: nitrogen incorporation can be confined to the channel region while leaving the contact regions in a degenerately doped, vacancy-rich state. As a result, Bi_2_O_2_Se field-effect transistors (FETs) are converted from depletion- to enhancement-mode operation with markedly improved electrostatic gate control, while maintaining ohmic, contact-transparent carrier injection. This decoupling of channel electrostatics from contact properties enables the simultaneous realization of high on/off ratios and band-like transport, mitigating a long-standing limitation in 2D FETs.

## Results and Discussion

### Chemical and Electronic States of N-Incorporated Bi_2_O_2_Se

We synthesized strain-free Bi_2_O_2_Se crystals by CVD under Se-deficient conditions, which minimize residual compressive strain by relieving interfacial lattice mismatch during cooling.^31^ Growth details are provided in the Methods. The resulting crystals adopt a highly symmetric tetragonal structure (*a* = *b* ≈ 3.88 Å, *c* ≈ 12.16 Å) consisting of alternating [Bi_2_O_2_]^2+^ and [Se]^2–^ layers stacked along the *c*-axis, characteristic of a non–van der Waals layered material (Supporting Information 1).^36^ The nitrogen incorporation was carried out by exposing the Bi_2_O_2_Se crystals to NH_3_ at 150 °C in a low-pressure chamber (∼200 mTorr; Figure 1a). To confirm that this process leads to vacancy passivation rather than random surface adsorption, we analyzed the adsorption energetics by DFT. The calculations show that an individual nitrogen atom preferentially chemisorbs at a surface V_Se_, forming three N–Bi bonds with an adsorption energy of −4.04 eV. This strong chemisorption confers robust thermodynamic stability, even under typical device processing temperatures, thereby establishing nitrogen as an effective passivating species for selenium vacancies. Nitrogen incorporation at subsurface sites is also energetically favorable (−3.95 eV), indicating that vacancy passivation is not limited to the surface. Such vacancy binding effectively passivates V_Se_ while preserving the integrity of the Bi_2_O_2_Se lattice. Bader charge analysis reveals substantial charge transfer from neighboring Bi atoms to nitrogen (1.28 e/atom), confirming strong N–Bi bonding and electron withdrawal consistent with acceptor-like behavior. This large local charge redistribution is consistent with strong N–Bi bonding and stabilization of the vacancy-passivated configuration, rather than with the formation of an isolated nitrogen-derived in-gap defect state (Figure 1b and Supporting Information 2).

**Figure 1:**
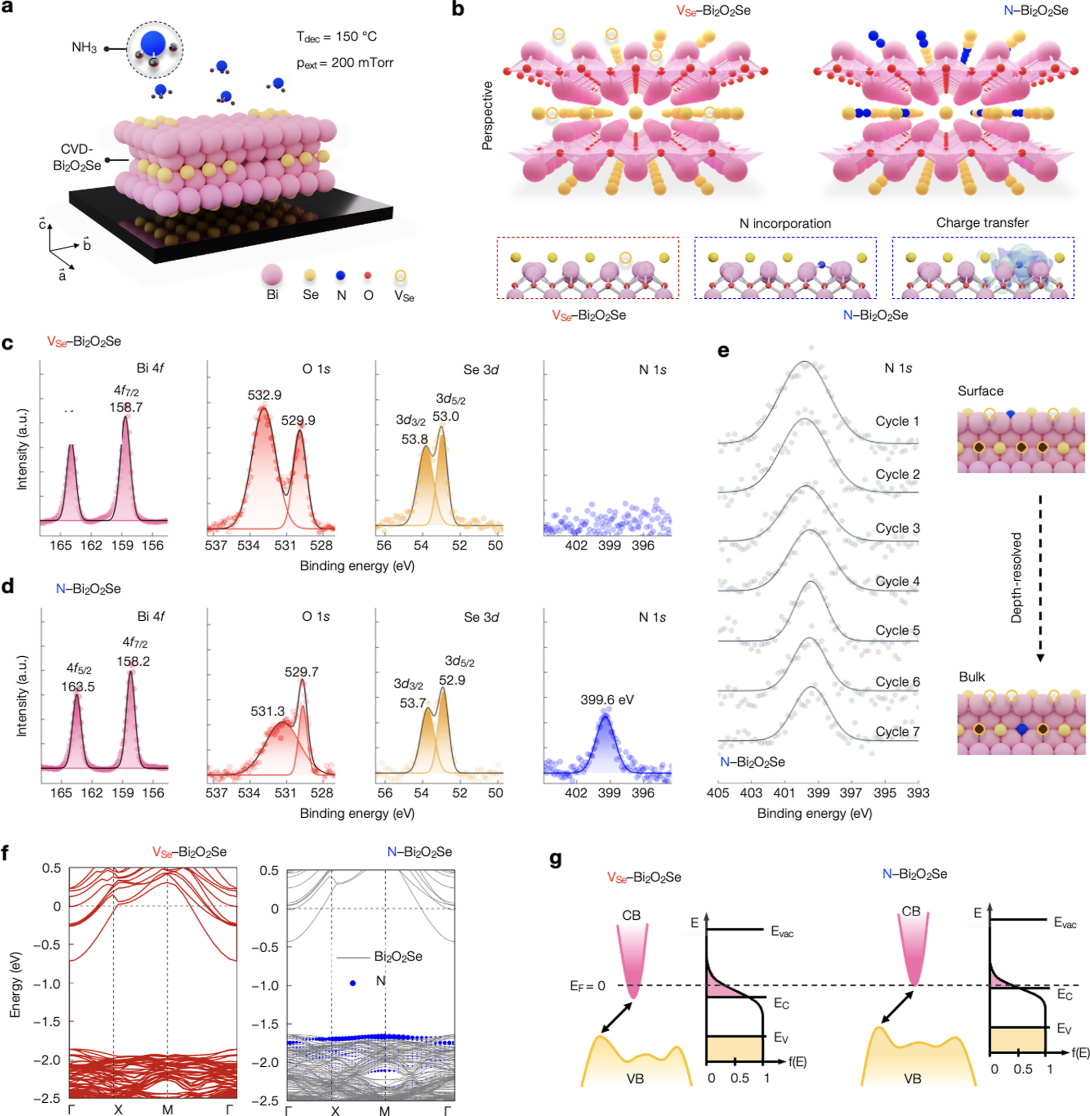
Electronic and chemical characterization of Se- and N–Bi_2_O_2_Se. (a) Schematic illustration of the postsynthesis annealing under NH_3_ atmosphere. (b) Structural schematics of V_Se_–Bi_2_O_2_Se and N–Bi_2_O_2_Se. Bottom panel shows the charge distribution induced by N incorporation. c,d, XPS spectra of Bi 4f, O 1s, Se 3d, and N 1s core levels acquired from V_Se_–Bi_2_O_2_Se and N–Bi_2_O_2_Se. (e) Depth-dependent XPS spectra of N 1s acquired from N–Bi_2_O_2_Se. (f) Electronic band structure of V_Se_–Bi_2_O_2_Se with 3.1% internal Se vacancies and N-incorporated Bi_2_O_2_Se. Dashed lines denote the Fermi levels, which are set to 0 eV; N-projected states are highlighted in blue. (g) Schematics for V_Se_–Bi_2_O_2_Se and N–Bi_2_O_2_Se Fermi–Dirac occupation, illustrating the redistribution of electronic occupation near the conduction band edges.

X-ray photoelectron spectroscopy (XPS) provides chemical evidence that nitrogen incorporation selectively passivates V_Se_ without inducing disruptive changes to the Bi_2_O_2_Se lattice (Figure 1c,d). In pristine films, the Bi 4f_5/2_ and Bi 4f_7/2_ core levels are located at 163.9 and 158.7 eV, respectively, while the Se 3d doublet appears at 53.8 and 53.0 eV. Following nitrogen treatment, a distinct N 1s signal emerges at 399.6 eV, whereas the Bi 4f and Se 3d binding energies remain unchanged, indicating preservation of the local chemical environment of the host lattice. Concurrently, the reduction of the O 1*s* component at 532.9 eV, associated with adsorbed oxygen, signifies effective passivation of surface vacancy sites.^37^ Depth-resolved XPS further reveals nitrogen incorporation to a depth of approximately 4 nm after 15 min of exposure (Figure 1e). A small positive shift in the bulk Se 3d peak suggests a higher residual vacancy density in the film interior, consistent with incomplete vacancy passivation at greater depths.

Electronic structure calculations (Figure 1f) show that nitrogen incorporation preserves the intrinsic band dispersion of Bi_2_O_2_Se and does not introduce midgap states near the conduction-band minimum (CBM). Instead of modifying the electronic bands, nitrogen passivation shifts the Fermi level (*E*_F_) downward toward the midgap, reflecting a reduced electron density while retaining delocalized conduction pathways. The corresponding Fermi–Dirac distributions (Figure 1g) illustrate this evolution: in V_Se_–Bi_2_O_2_Se, the occupation probability extends into the conduction band, whereas in N-incorporated Bi_2_O_2_Se (N– Bi_2_O_2_Se), charge distribution decays exponentially above *E*_F_, consistent with carrier statistics shifting toward a nondegenerate regime.^38^

### Local Electronic Structure of V_Se_- and N–Bi_2_O_2_Se

STM/STS were employed to probe the atomic structure and local density of states (LDOS) of Bi_2_O_2_Se before and after N incorporation (Figure 2a–c). The differential conductance (d*I*/d*V*), which reflects the LDOS, revealing a clean bandgap with no observable in-gap states for both V_Se_- or N–Bi_2_O_2_Se. To further assess the electronic impact of N incorporation, we calculated the total (Figure 2d) and element-resolved DOS (Supporting Information 4) for a 1.6% N-incorporated configuration, the band edges remain intact while the Fermi level moves toward the midgap, consistent with reduced carrier concentration. In pristine V_Se_–Bi_2_O_2_Se, self-doping elevates the Fermi level into the conduction band, resulting in degenerate *n*-type behavior. Upon N adsorption, electrons are withdrawn from donor vacancies, lowering the chemical potential and reducing free-electron density. Nitrogen compensation in Bi_2_O_2_Se is best understood as vacancy neutralization rather than the formation of a conventional nitrogen-derived acceptor level. The projected density of states shows that N does not introduce new localized midgap states, nor additional localized states immediately below the conduction-band minimum. Instead, nitrogen suppresses the donor-like contribution associated with selenium-vacancy-related defect environments. Kelvin probe force microscopy (KPFM, Supporting Information 5) further confirms this behavior, showing an increased surface potential after N incorporation.

**Figure 2:**
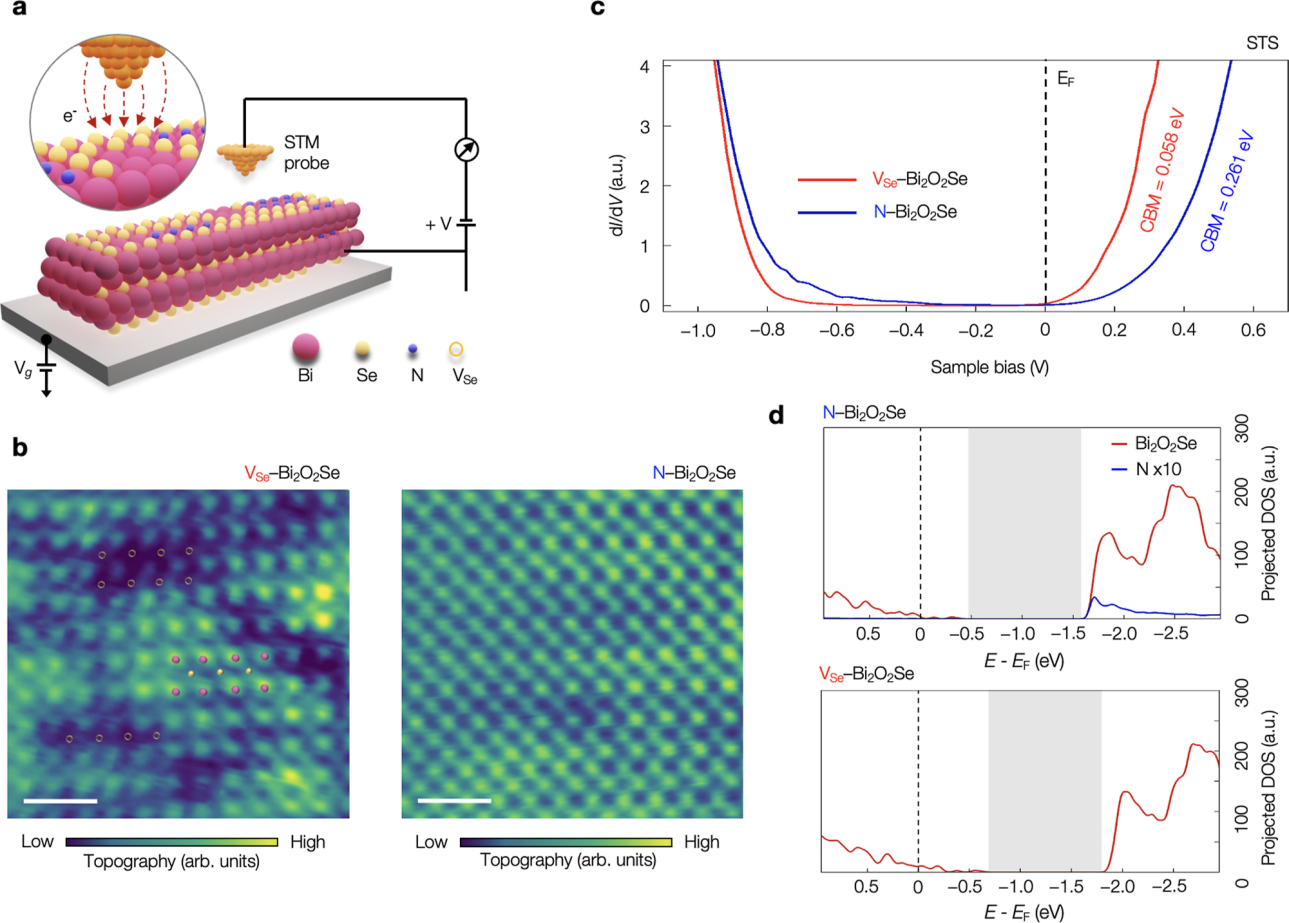
STM/STS measurements of V_Se_–Bi_2_O_2_Se and N–Bi_2_O_2_Se. (a) Experimental configuration for STM/STS measurements, with Bi_2_O_2_Se placed on a HOPG substrate. (b) High-resolution STM topographies of V_Se_–Bi_2_O_2_Se and N–Bi_2_O_2_Se, revealing the disappearance of characteristic Se-vacancy dimer features following N incorporation (scale bar: 1 nm). (c) Representative d*I*/d*V* spectra of V_Se_–Bi_2_O_2_Se and N–Bi_2_O_2_Se, showing the absence of detectable midgap states. (d) Calculated density of states for Bi_2_O_2_Se containing 3.1% internal Se vacancies, and for Bi_2_O_2_Se with 1.6% N concentration. The gray-shaded region denotes the bandgap of pristine Bi_2_O_2_Se. The Fermi level is set to 0 eV.

To visualize the corresponding atomic-scale modifications, we compared STM topographies of V_Se_- and N–Bi_2_O_2_Se (Figure 2b). In the pristine material, V_Se_ appear as characteristic dimerized defect pairs, consistent with previous reports.^21^ DFT calculations show that nitrogen preferentially occupies these dimer sites, forming N–Bi bonds that energetically passivate the vacancies. Following N incorporation, the characteristic dimer features vanish from STM images, consistent with effective vacancy passivation. Statistical analysis over large-area scans (Supporting Information 6) reveals substantial reductions in both the average dimer length and defect density. Taken together with spectroscopic evidence, these microscopic observations support that N incorporation selectively passivates donor-type vacancies, shifts the Fermi level toward midgap, and preserves the structural coherence of Bi_2_O_2_Se.

### Electrical Characteristics of Bi_2_O_2_Se FETs

N-induced compensation of donor-type V_Se_ enhances electrostatic gate control in Bi_2_O_2_Se. To evaluate this effect, we fabricated back-gated FETs using degenerately doped V_Se_–Bi_2_O_2_Se channels on SiO_2_/p^++^–Si substrates (Figure 3a,b), followed by postgrowth nitrogen treatment at 150 °C for 5 min (predominantly surface incorporation) or 15 min (bulk incorporation). Pristine V_Se_–Bi_2_O_2_Se FETs exhibit degenerate *n*-type transport with weak gate modulation, consistent with a high electron density originating from V_Se_ donors. After N incorporation, the devices transition to semiconducting operation with markedly improved gate control (Figure 3c), indicating effective suppression of excess electron density. Compared with surface treatment, bulk N incorporation yields a larger improvement in tunability, consistent with deeper penetration and more uniform passivation of donor vacancies throughout the channel. The transfer characteristics (Figure 3d) show a systematic threshold-voltage (*V*_th_) shift from −16.7 V in V_Se_–Bi_2_O_2_Se (depletion mode) to +9.6 V in N–Bi_2_O_2_Se (enhancement mode), whereas the V_Se_–Bi_2_O_2_Se FET can only be turned off under a large *V*_GS_ of ∼−30 V. The full output-characteristic measured over *V*_GS_ = −10 to 10 V is shown in Supporting Information 7.

**Figure 3:**
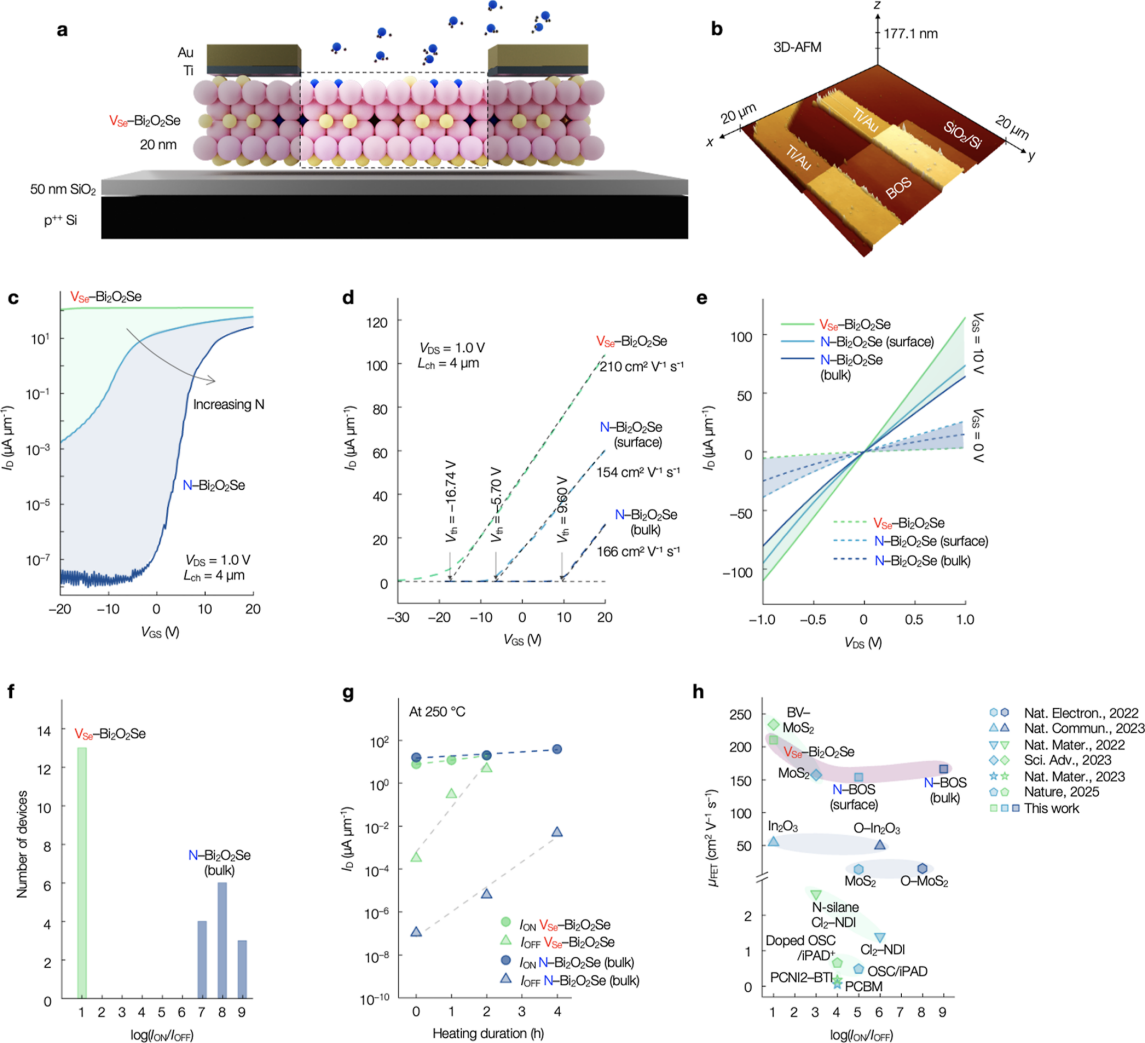
Electrical characteristics of back-gated Bi_2_O_2_Se FETs before and after N incorporation. (a) Schematic illustration of FET configuration. (b) 3D AFM image of a Bi_2_O_2_Se FET fabricated on a 50 nm SiO_2_/p^++^–Si substrate, with channel thickness of 20 nm. (c,d) Transfer characteristics of Bi_2_O_2_Se FETs with different levels of N incorporation, showing the evolution from weakly gated, degenerate transport to gate-controllable semiconducting operation. (e) Output characteristics of the devices measured at *V*_GS_ = 0 V and *V*_GS_ = 20 V. (f) Statistical distribution of the on/off current ratio (*I*_ON_/*I*_OFF_) extracted from 13 devices before and after N incorporation. g, Thermal stability of V_Se_–Bi_2_O_2_Se and N–Bi_2_O_2_Se FETs in prolonged heating at 250 °C. (h) Benchmarking of μ_FET_ versus *I*_ON_/*I*_OFF_ for representative 2D FETs reported in literature. Devices employing *n*-type and *p*-type doping strategies are indicated by green- and gray-shaded regions, respectively, with dopant species labeled.

This electrostatic transition is accompanied by substantial improvements in overall device performance. Bulk N incorporation yields a high electron mobility of 166 cm^2^ V^–1^ s^–1^ and an on/off current ratio approaching 10^9^, indicating efficient gate modulation while maintaining low defect-induced scattering. The simultaneous realization of high mobility and strong gate control, which are typically competing metrics in 2D transistors, highlights the advantage of selective vacancy passivation through defect engineering. Output characteristics measured at *V*_GS_ = 0 and 10 V (Figure 3e) exhibit linear *I*–*V* behavior and enhanced low-bias current injection, consistent with improved contact transparency following N incorporation.

Statistical analysis of 13 devices (Figure 3f) shows excellent reproducibility, with consistent *V*_th_ shifts and mobility enhancement across samples, indicating the robustness of N incorporation process. Thermal stressing at 250 °C promotes the activation of V_Se_, resulting in an increased on-state conduction but simultaneously elevates the off-state leakage, and hence a degradation of the on/off ratio (Figure 3g and Supporting Information 8). Notably, the N-incorporated devices exhibit a much smaller increase in *I*_OFF_ under the same stress, indicating that N incorporation mitigates vacancy-induced leakage and improves thermal robustness.

The generality of this passivation strategy is further validated across different dielectric environments and channel thicknesses (Supporting Information 9), where similar electrostatic trends are consistently observed. Benchmarking against previously reported 2D semiconductor FETs (Supporting Information Tables S1 and S2 and Figure 3h) reveals a fundamental trade-off: conventional *n*-type doping enhances conductivity at the expense of gate control, whereas *p*-type doping improves electrostatic modulation but degrades mobility. In contrast, bulk nitrogen incorporation in Bi_2_O_2_Se reconciles these competing effects, simultaneously achieving high field-effect mobility and strong gate tunability.

### Temperature-Dependent Transport Regimes

To elucidate how nitrogen defect engineering modulates intrinsic charge transport, we investigated the temperature-dependent behavior of Bi_2_O_2_Se FETs with selective and full N incorporation. Channel-selective N incorporation enables precise electrostatic modulation of the channel while preserving degenerately doped V_Se_–Bi_2_O_2_Se contacts, thereby suppressing Schottky-barrier effect and maintaining ohmic carrier injection. This controlled device configuration provides a platform for distinguishing contact-limited from channel-limited transport, an essential requirement for complementary logic integration (Supporting Information 10–12). Two device geometries were compared: (i) channel-selectively N-incorporated FETs, in which only the channel region is compensated while the contacts remained degenerate, and (ii) fully N-incorporated FETs, in which both channel and contact regions are compensated. For devices with degenerate contacts, *I*_D_–*V*_GS_ measurements (Figure 4a) reveal a crossover in the temperature dependence of conductivity, indicative of a gate-induced metal–insulator transition (MIT). At low *V*_GS_, transport is insulating and thermally activated, whereas at high *V*_GS_ it evolves into band-transport behavior, consistent with a gate-driven Fermi level shift across a mobility edge.^39^ In contrast, such intrinsic transport signatures are absent in contact-limited devices, where Schottky barriers dominate carrier injection and obscure channel conduction. Once contact transparency is ensured, the observed crossover emerges as a manifestation of intrinsic channel physics.^40^

**Figure 4:**
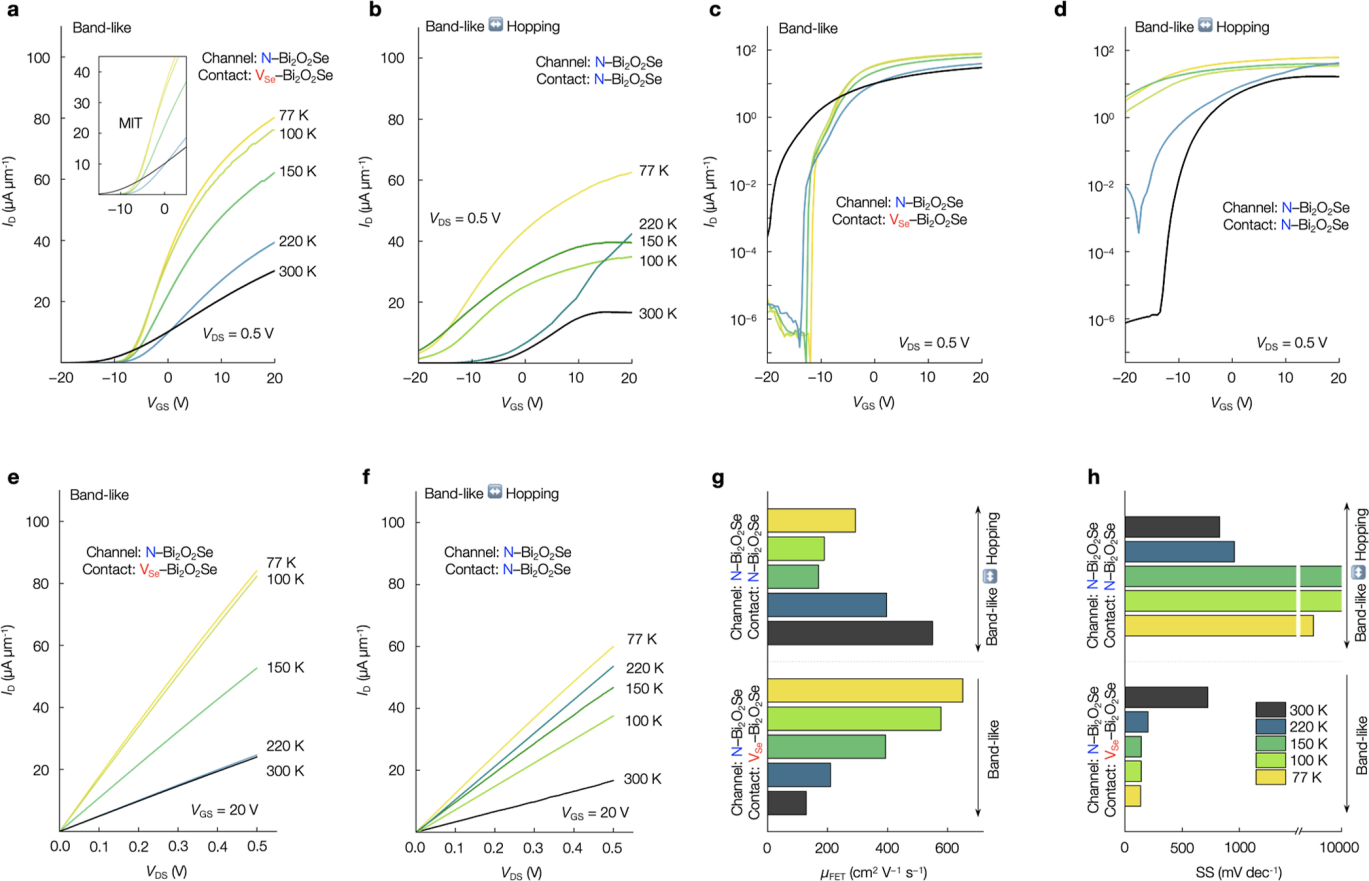
Temperature-dependent transport in Bi_2_O_2_Se FETs. (a,b) Transfer characteristics of Bi_2_O_2_Se FETs with a N-incorporated channel and either V_Se_–Bi_2_O_2_Se contacts (a) or N–Bi_2_O_2_Se contacts (b), measured at *V*_DS_ = 0.5 V. (c,d) Corresponding transfer characteristics in logarithmic scale. (e,f) Output characteristics, measured at *V*_GS_ = 20 V for devices with V_Se_–Bi_2_O_2_Se contacts (e) and N–Bi_2_O_2_Se contacts (f). (g) Temperature-dependent μ_FET_ across the band-like–to–hopping transport crossover. (h) Comparison of subthreshold swing (SS) as a function of temperature. Band-like devices approach the thermal limit and exhibit approximately linear scaling with temperature, while hopping-dominated devices display broadened SS and nonlinear temperature dependence.

Arrhenius analysis of ln­(*I*_DS_/*T* ^1.5^) versus 1/*T* exhibits linear behavior with negative slopes (Supporting Information 13a), indicating that carrier injection is not limited by thermionic emission over a Schottky barrier. This behavior is consistent with degenerate V_Se_–Bi_2_O_2_Se contacts maintaining ohmic, transparent injection down to low temperatures, in line with their degenerate carrier statistics (Figure 4a,c). In contrast, fully N-incorporated devices display a pronounced gate-dependent crossover between band-like transport and localized conduction, characteristic of Fermi level modulation within the channel (Figure 4b,d). When N incorporation extends into the contact region, the Fermi level shifts deeper into the bandgap, leading to the formation of a depletion region that impedes carrier injection. Below approximately 150 K, transport becomes increasingly dominated by hopping through localized interfacial states and/or tunneling across the depletion barrier, giving rise to the observed strong temperature dependence of the drain current.

Further evidence of these distinct transport regimes is provided by the *I*_D_–*V*_DS_ characteristics and temperature-dependent mobility trends. In the band-like regime, the *I*_D_–*V*_DS_ curves remain linear and the mobility increases upon cooling (Figure 4e,g), consistent with reduced phonon scattering. In devices exhibiting a crossover from band-like to localized transport, the drain current becomes strongly temperature-dependent below approximately 150 K (Figure 4f and Supporting Information 14). Channel-selectively N-incorporated devices show only a modest reduction in conductance with decreasing temperature, consistent with transport through extended electronic states, whereas fully N-incorporated devices display pronounced conductance degradation indicative of carrier localization.^41^ Mobility and subthreshold swing (SS) analyses further reinforce this distinction: in channel-selectively N-incorporated FETs, the mobility increases with decreasing temperature, while fully incorporated devices exhibit thermally activated mobility below 150 K (Figure 4g). Correspondingly, the SS sharpens under band-like transport but broadens in the hopping-dominated regime (Figure 4h), reflecting weakened gate coupling in the presence of localized states.

Collectively, these observations delineate a clear doping- and temperature-dependent transport crossover boundary separating band-like and hopping regimes in N–Bi_2_O_2_Se. Selective nitrogen incorporation preserves degenerate contacts and enables access to the intrinsic band transport of the channel, whereas full incorporation promotes carrier localization at compensated interfaces. This ability to spatially engineer transport regimes demonstrates that selective defect engineering provides a route for designing contact-transparent and low-power 2D logic devices.

## Conclusions

This work demonstrates a selective defect-engineering strategy that transforms Bi_2_O_2_Se from a self-doped conductor into a tunable semiconductor. Nitrogen incorporation passivates selenium vacancies while preserving the intrinsic band dispersion and avoiding the introduction of midgap trap states, enabling controlled modulation of carrier density without compromising contact transparency. The resulting Bi_2_O_2_Se transistors simultaneously achieve high electron mobility and strong electrostatic gate control, exhibiting a clear electrostatic transition from depletion- to enhancement-mode operation. Crucially, the spatial selectivity of this process enables channel-specific compensation while maintaining degenerately doped, quasi-ohmic contacts, thereby providing access to the intrinsic band-like transport of a 2D oxide semiconductor. Because nitrogen incorporation is decoupled from crystal growth and performed under mild thermal conditions, this approach is potentially scalable and compatible with wafer-level device processing. Together, these findings establish selective defect engineering as a versatile framework for realizing contact-transparent, gate-controllable 2D transistors, bridging atomic-scale defect control with device-level functionality for next-generation low-power logic electronics.

## Methods

### Material Synthesis and Transfer

2D Strain-free Bi_2_O_2_Se crystals were synthesized using a custom-built chemical vapor deposition (CVD) system consisting of 90 cm horizontal tube furnaces with 1-in.-inner-diameter quartz tubes. Bi_2_O_3_ (99.999%, Alfa Aesar) and Bi_2_Se_3_ (99.999%, Alfa Aesar) powders served as coevaporation sources. One cm × 1 cm cleaved mica substrate was placed above the overlapping Bi_2_O_3_ and Bi_2_Se_3_ source region, with a spacing of 0.3 cm. The precursors were arranged in an alternating checkerboard-like pattern on the quartz boat beneath the substrate to promote a more uniform local vapor environment during growth. The deposition was carried out at a total pressure of 400 Torr under a 200 sccm flow of Ar. The furnace was heated to 580 °C and rapidly cooled to room temperature after deposition. 2D Bi_2_O_2_Se crystals were transferred using polydimethylsiloxane (PDMS). During the transfer process, a custom-built mechanical platform was used to slightly press the mica/PDMS stack onto the target substrate, releasing a strain-free Bi_2_O_2_Se layer onto the surface. The samples were then annealed at 150 °C for 1 h to strengthen adhesion.

### Nitrogen Treatment of Bi_2_O_2_Se

Nitrogen incorporation was performed by exposing Bi_2_O_2_Se crystals to NH_3_ at 150 °C in a low-pressure chamber (∼200 mTorr). Bi_2_O_2_Se flakes were subjected to nitrogen treatment for 5 or 15 min. The shorter 5 min exposure mainly produced surface nitrogen incorporation, whereas the longer 15 min exposure enabled more extensive incorporation beyond the surface, which is required for effective incorporation of selenium-vacancy-related defects in the bulk/channel region.

### Device Fabrication and Measurement

A heavily doped Si wafer (resistivity 0.001–0.005 Ω cm) with a 50 nm thermally grown SiO_2_ layer served as the gate electrode and dielectric. Bi_2_O_2_Se channel layers were transferred onto SiO_2_ using the procedure described above. A channel length of 4 μm for field-effect transistors (FETs) was patterned with digital light processing (DLP) lithography, unless otherwise mentioned. Ti/Au (10/30 nm) were evaporated using electron-gun in high vacuum to form source/drain electrodes. Electrical measurements were performed at room temperature using a Keithley 4200 SCS.

The field-effect mobility μ_FE_ was determined from the transconductance in the linear regime of the transfer characteristics according to

$$\mu_{FE}=\frac{L}{WC_{ox}V_{DS}}g_{m}$$

where *L* and *W* are the channel length and width, *V*_DS_ is the applied drain-source bias, and *g*_m_ = ∂*I*_DS_/∂*V*_GS_ is the transistor conductance. The gate-oxide capacitance per unit area was calculated from

$$C_{ox}=\frac{\varepsilon_{0}\varepsilon_{r}}{t_{ox}}$$

using ε_0_ = 8.854 × 10^–12^ F m^–1^, ε_r_ = 3.9 for SiO_2_, and an oxide thickness *t*_ox_ = 50 nm, giving *C*_ox_ ≈ 6.9 × 10^–4^ F m^–2^. Mobility values are reported in cm^2^ V^–1^ s^–1^.

### Material Characterization and Measurements

Binding energy analysis was carried out by XPS (Thermo Fisher). The thickness of the layer-by-layer Bi_2_O_2_Se films was determined using tapping-mode atomic force microscopy (AFM; Bruker Dimension Icon). Surface potential measurements were performed using Kelvin probe force microscopy (KPFM) in ambient conditions on an atomic force microscope (AFM; Bruker Dimension Icon) operated in tapping/lift mode. STM/STS experiments were conducted at room temperature (∼295 K). Tungsten tips were prepared through ex situ electrochemical etching. Topographic measurements were obtained in constant current mode, with bias voltages applied to the sample with respect to the STM tip. The band gaps from STS were extracted by linearly fitting the valence- and conduction-band edges and identifying the band gap minimum in the log­(d*I*/d*V*) spectrum.

### First-Principles Calculations

First-principles calculations based on density functional theory were performed to investigate the adsorption behavior of N on Bi_2_O_2_Se. All simulations were carried out using the Vienna Ab initio Simulation Package (VASP),^42^ employing the generalized gradient approximation (GGA) in the form proposed by Perdew, Burke, and Ernzerhof (PBE).^43^ The interactions between core and valence electrons were described using the projector augmented-wave (PAW) method,^44^ and a plane-wave energy cutoff of 600 eV was adopted.

To model the Bi_2_O_2_Se film, a slab consisting of three Bi_2_O_2_Se chemical layers was constructed, with both surfaces terminated by a 50% selenium coverage with 50% Se-vacancy dimers, in alignment with experimentally characterized cleavage surfaces. In order to capture the degenerate n-type characteristics arising from selenium vacancies (V_Se_) in CVD-grown Bi_2_O_2_Se, two inner-layer Se atoms were removed, corresponding to an effective vacancy concentration of 3.1%. The in-plane lattice constant was set to 15.52 Å, and a vacuum region exceeding 18 Å was included along the out-of-plane direction to prevent artificial interactions between periodic images. This configuration with internal Se vacancies was employed to simulate the adsorption of a single nitrogen atom at a surface Se vacancy site, corresponding to an adsorption coverage of 1.6%. To avoid spurious interactions between adsorbates, an interatomic spacing of 15.52 Å was maintained between nitrogen atoms. Structural optimizations were performed until the residual forces on all atoms were less than 0.01 eV/Å, and the total energy converged to within 1 × 10^–5^ eV per supercell. Calculations were carried out self-consistently using a Monkhorst–Pack *k*-point mesh of 2 × 2 × 1, which corresponds to a *k*- spacing of 0.03 (2π/Å).

## Supplementary Material



## Notes

Crystal structure of CVD-grown Bi_2_O_2_Se. Charge transfer characteristics of N–Bi2O2Se. Element-resolved density of states (DOS). Surface potential mapping. Statistical analysis over large-area STM. The full voltage sweep *I*_D_–*V*_DS_ (−10 to 10 V). The generality of this passivation strategy. Monolithic 3D integration of BEOL-compatible Bi2O2Se on Si FinFET platforms. Rigorous quantification of contact transparency. (PDF)
