# Supplementary material for: Selective Defect Engineering for Gate-Controlled yet Contact-Transparent Bi2O2Se Transistors
Source: ACS Nano. 2026 May 26;20(25):18315–23. doi: 10.1021/acsnano.6c04248 (PMC13325869; doi:10.1021/acsnano.6c04248)
Supplement: Supplementary file 1 [file nn6c04248_si_001.pdf]

Supporting Information

Selective Defect Engineering for Gate-Controlled yet Contact-Transparent Bi_2O_2Se Transistors

Huynh-Uyen-Phuong Nguyen,[1] Tai-Ting Lee,[3,4] Yu-Wei Chang,[3] Hung-Chang Hsu,[3] Chih-Yuan Shih,[3] Chi-Chun Cheng,[2] Luc-Phuong-Nhu Tran,[2] Hsin-Chien Chien,[1] Wen-Yuan Fei,[1] Wei-Yen Woon,[5] Yung-Chang Lin,[6] Kazu Suenaga,[7] Yen-Fu Lin,[8] Ya-Ping Chiu,[3] Mei-Yin Chou,[3,4] Po-Wen Chiu[1,2]

[1]College of Semiconductor Research, National Tsing Hua University, Hsinchu, Taiwan

[2]Department of Electrical Engineering, National Tsing Hua University, Hsinchu, Taiwan

[3]Department of Physics, National Taiwan University, Taipei, Taiwan

[4]Institute of Atomic and Molecular Sciences, Academia Sinica, Taipei, Taiwan

[5]Corporate Research, Research and Development, Taiwan Semiconductor Manufacturing Company (TSMC), Hsinchu, Taiwan

[6]Nanomaterials Research Institute, National Institute of Advanced Industrial Science and Technology (AIST), Tsukuba, Japan

[7]The Institute of Scientific and Industrial Research (ISIR-SANKEN), Osaka University, Osaka, Japan.

[8]Department of Physics, National Chung Hsing University, Taichung, Taiwan

Crystal structure of CVD-grown Bi_2O_2Se.

Supplementary Figure 1 provides structural confirmation of the Bi_2O_2Se crystal phase and the high crystalline quality of the 2D channel used in this work. Bi_2O_2Se crystallizes in a body-centered tetragonal structure (space group *I4/mmm*), with an in-plane square lattice ($a = b$) and a larger out-of-plane lattice constant (c) reflecting its layered stacking. Reported lattice parameters are typically $a = b \approx 3.88$ Å and $c \approx 12.16$ Å. The structure can be viewed as alternating positively charged Bi_2O_2 layers and negatively charged Se layers along the c-axis, which underpins the quasi-2D nature of the material and motivates its use as a thin, high-mobility semiconductor. The FFT extracted from the cross-sectional STEM image exhibits sharp, discrete diffraction spots evidencing long-range crystallographic order and a well-defined orientation across the field of view. The spot symmetry and spacing are consistent with a tetragonal lattice and corroborate that the observed periodic stacking in (b) arises from the intrinsic Bi_2O_2Se crystal structure

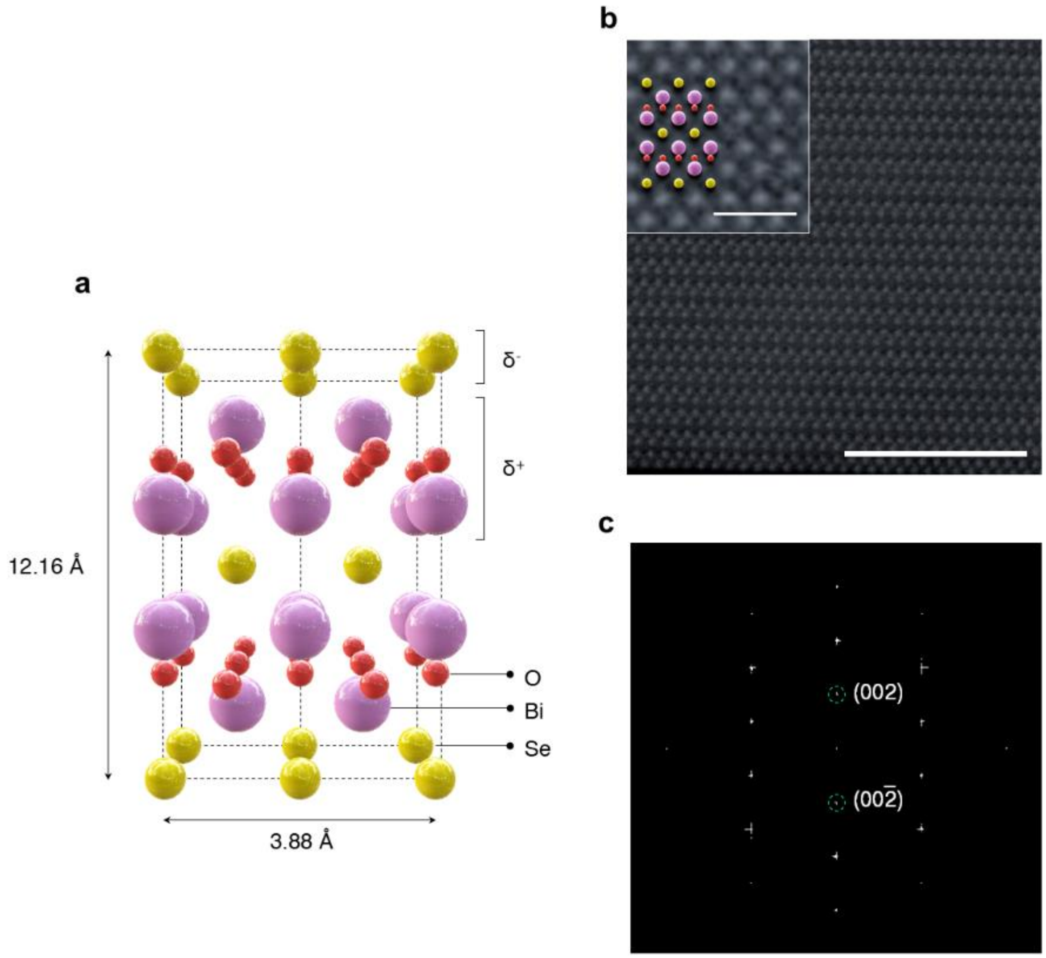

Supplementary Figure 1. a, Crystal structure and lattice constants of Bi_2O_2Se. **b,** Cross-sectional high-angle annular dark field scanning transmission electron microscopy (HAADF-STEM) image of a single-crystalline 2D Bi_2O_2Se, scale bar: 5 nm. **c,** FFT pattern of of **(b)**.

Charge transfer characteristics of N–Bi_2O_2Se.

To gain insight into the effect of N adsorption on Bi_2O_2Se, we have performed first-principles calculations to investigate the induced changes in electronic properties. A slab consisting of three chemical Bi_2O_2Se layers is used to model the film. The film terminates at the Se layer on both sides, each with a 50% Se coverage in a 4×4 supercell. This configuration ensures a net zero polarization in the film consisting of charged constituent layers. Intrinsic Se vacancies are commonly observed in CVD-grown Bi_2O_2Se films, giving rise to a carrier concentration characteristic of an n-type degenerate semiconductor. We simulate this behavior by removing two Se atoms in the inner layers per supercell. To study nitrogen adsorption, a single N atom is introduced into the surface supercell. Our calculated results reveal that the N atom preferentially adsorbs at a surface Se vacancy (V_{Se}) site, as shown in Supplementary Figure 2. This finding agrees with previous experimental observations and theoretical results, which identified surface Se vacancies as active centers for molecular adsorption. In the most favorable configuration, the adsorbed N atom forms strong chemical bonds with three adjacent Bi atoms. Bader charge analysis confirms a substantial charge transfer of 1.28 e per N atom from the substrate to the adsorbate, indicating a strong chemical interaction.

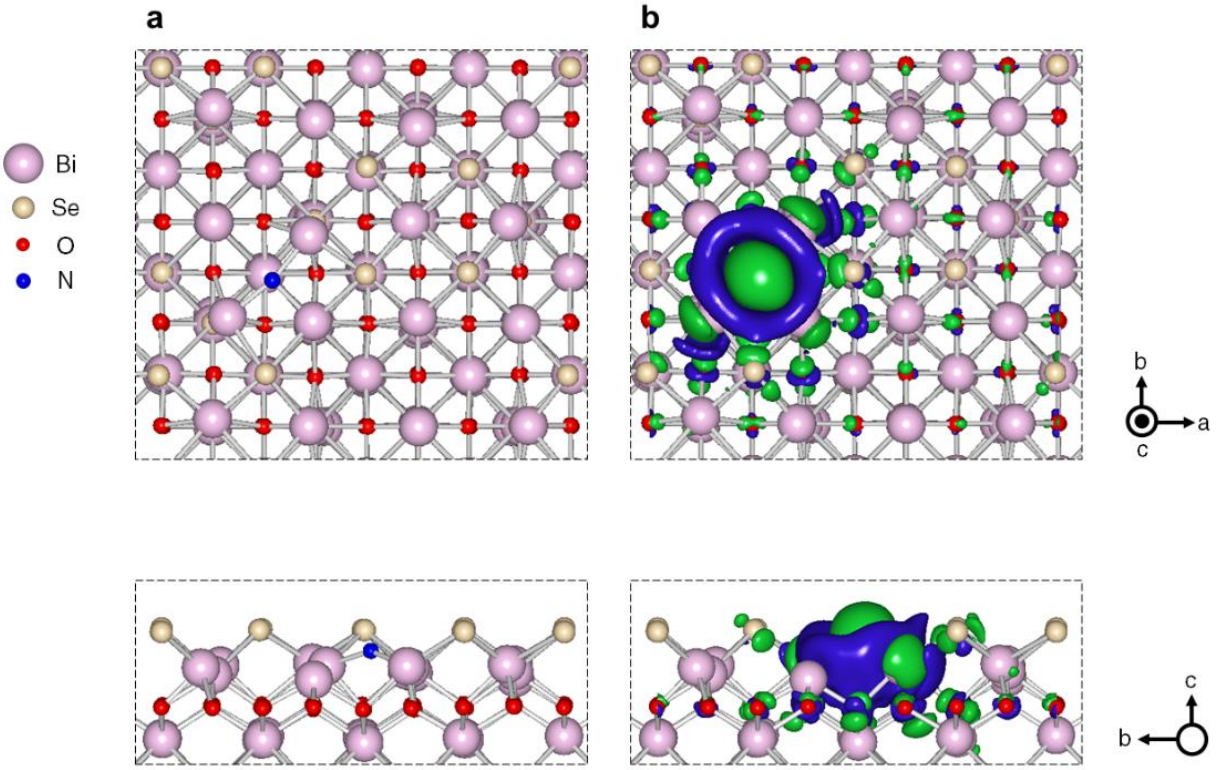

Supplementary Figure 2. Charge transfer characteristics of N-Bi_2O_2Se. **a**, Top and side views of the optimized configuration of nitrogen adsorption on the surface. **b**, Charge density change after adsorption for the configuration in (**a**). The green and blue regions represent electron accumulation and depletion, respectively.

Chemical characteristics of N–Bi_2O_2Se (bulk).

Compared with the surface-doped case, bulk doping typically yields a comparative robust and persistent N 1s signal, reflecting incorporation beyond the immediate surface. Concomitantly, the Se 3d core level may show a slight chemical shift consistent with a more extended electronic perturbation (Supplementary Figure 3). The key point is the *relative* behavior: bulk incorporation is supported when the N 1s intensity remains substantial even after accounting for surface sensitivity.

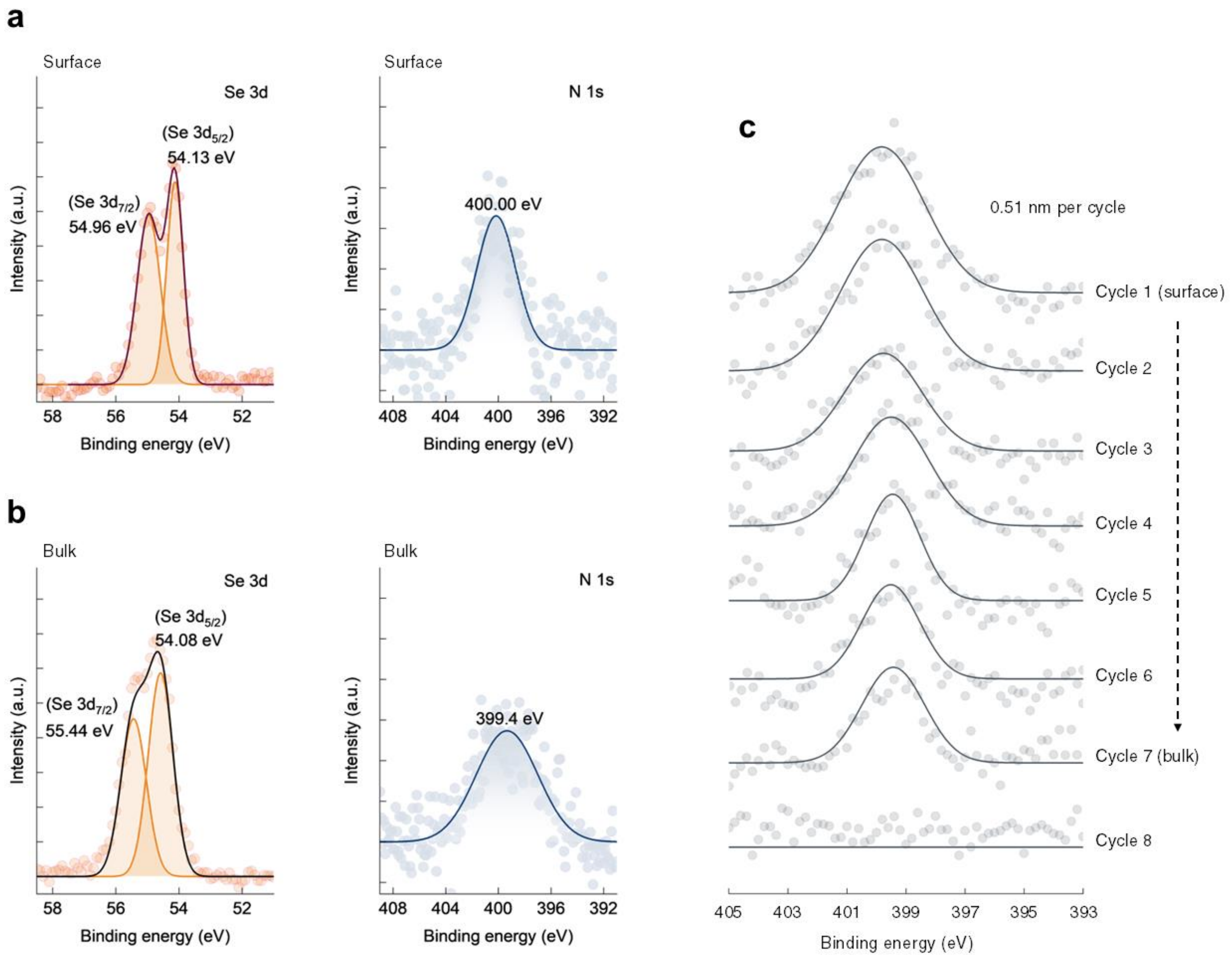

Supplementary Figure 3. a,b, XPS spectra of Se 3*d*, and N 1*s* core levels acquired from N-Bi_2O_2Se after 15 minutes of doping on the surface **(a)** and bulk **(b)**. **c,** Depth-resolved XPS spectra revealing variations in N 1s in N-Bi_2O_2Se (bulk).

Element-resolved density of states (DOS).

Supplementary Figure 4 shows the element-resolved density of states of the Bi_2O_2Se film with 3.1% internal Se vacancies, and the 1.6% N-adsorbed system. Dashed black lines indicate the chemical potential of each system, and bandgap regions are shaded gray. All energies are aligned to the vacuum level.

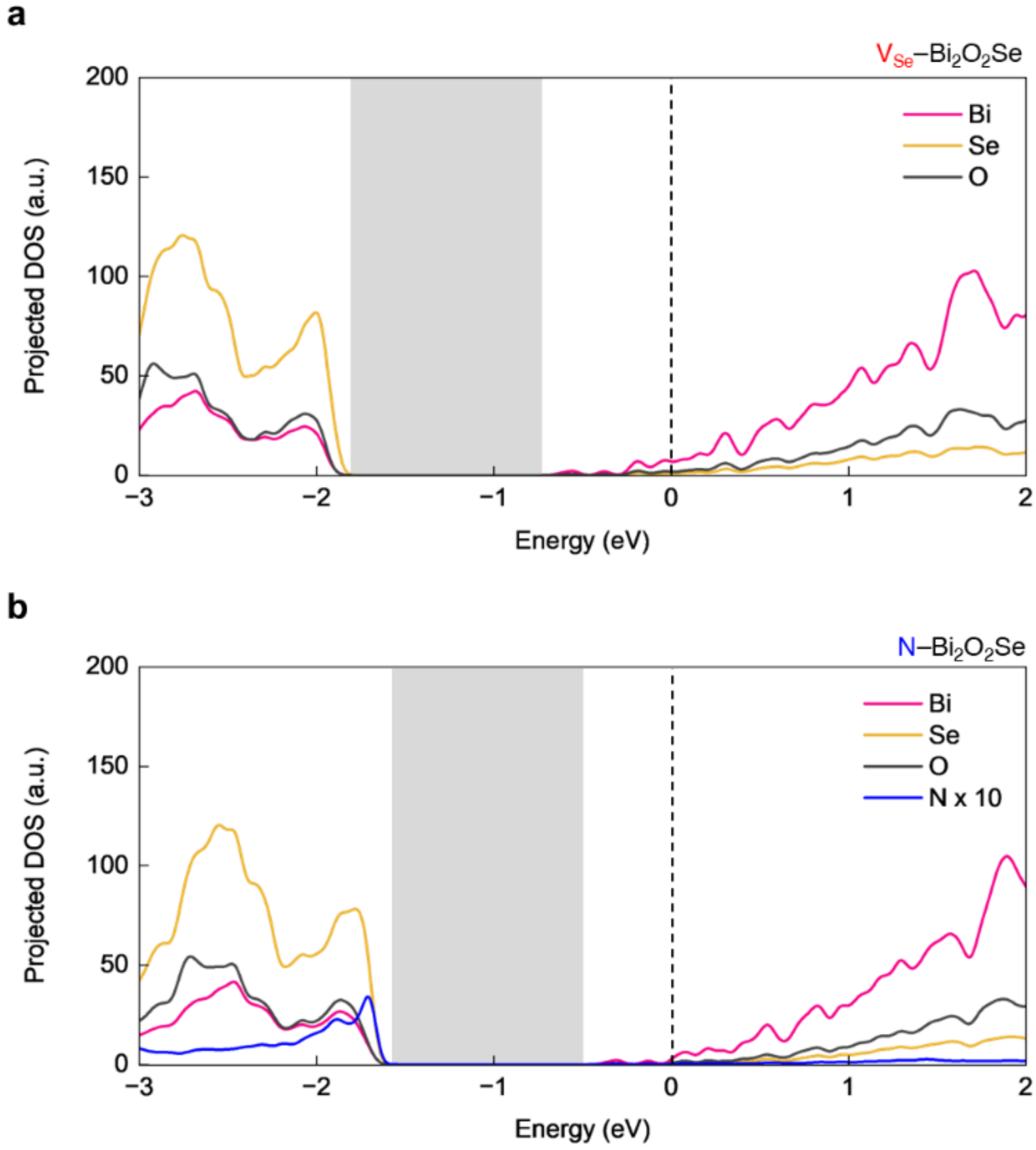

Supplementary Figure 4. a, Projected density of state (PDOS) showing the contributions of Bi, O and Se in V_{Se}-Bi_2O_2Se, with 3.1% internal Se vacancies. **b,** PDOS showing the contributions of Bi, O Se and N in N-Bi_2O_2Se with a 1.6% N system. Fermi level is set to 0 eV.

Surface potential mapping.

We examined the surface potential of pristine Bi_2O_2Se before and after nitrogen incorporation (Supplementary Fig. 5). The measurements show pronounced change in the surface-potential after nitrogen incorporation.

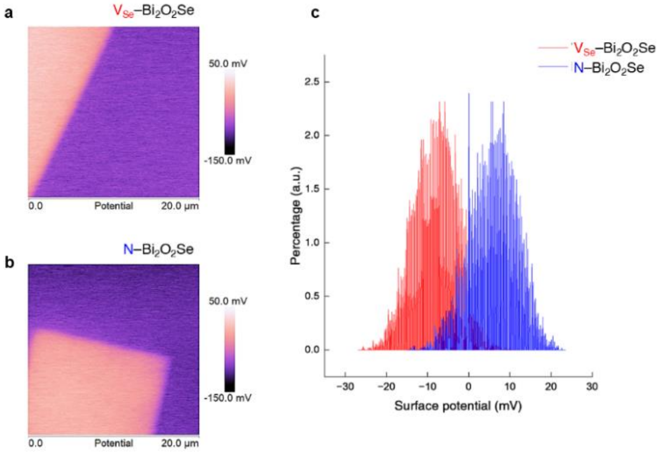

Supplementary Figure 5. a,b, Kelvin Probe Force Microscope (KPFM) analysis revealing surface potential variation in V_{Se}-Bi_2O_2Se **(a)** and N-Bi_2O_2Se **(b)**. **c,** Histogram of surface potential distribution obtained from KPFM measurements of V_{Se}-Bi_2O_2Se and N-Bi_2O_2Se.

STM topography.

Supplementary Figure 6 shows the large-scale atomic-resolution STM topography maps of the surface of V_{Se}-Bi_2O_2Se (a) and N-Bi_2O_2Se (b), in which Se vacancies dimerize and exhibit 2 × *n* structures (where *n* is an integer).

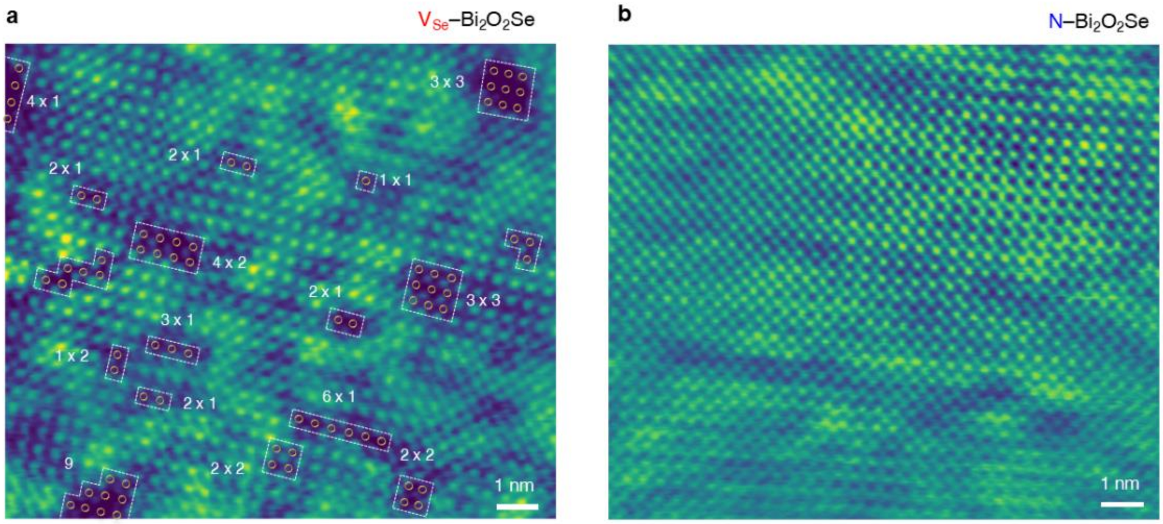

Supplementary Figure 6. a,b, Large-scale STM image of the surface of V_{Se}-Bi_2O_2Se **(a)** and N-Bi_2O_2Se **(b)**.

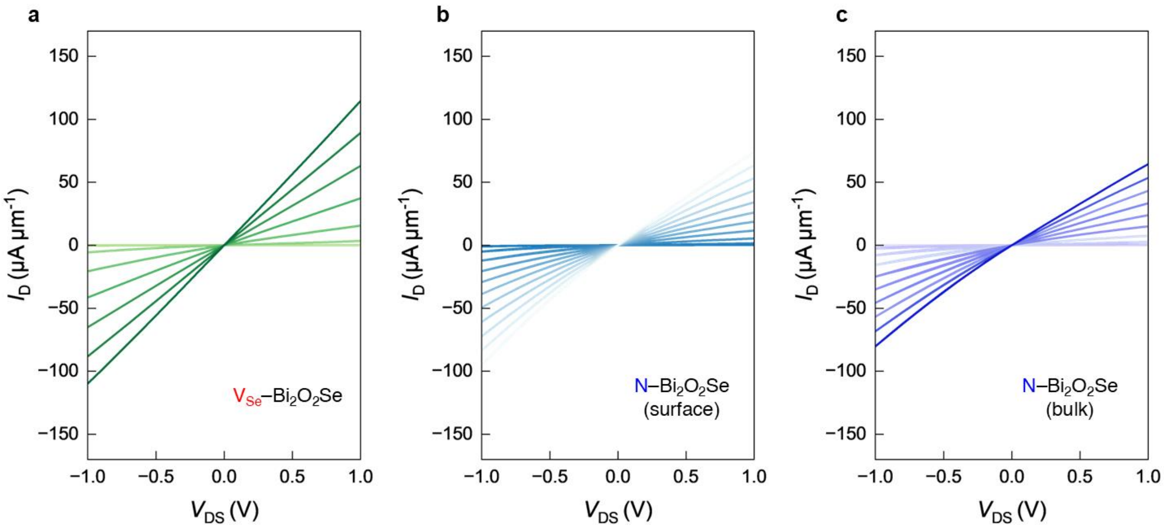

Supplementary Figure 7. a, Output characteristics (I_D–V_{DS}) curves of a Bi_2O_2Se FET device with a degenerate channel V_{Se}-Bi_2O_2Se. **b,c,** Output characteristics (I_D–V_{DS}) curves of a Bi_2O_2Se FET device with a N-Bi_2O_2Se channel for surface adsorption **(b)** and bulk adsorption **(c)**. V_{GS} sweeping range is from −10 to 10V.

Device performance of a degenerate channel before and after nitrogen adsorption under heating at 250 °C for an extended period.

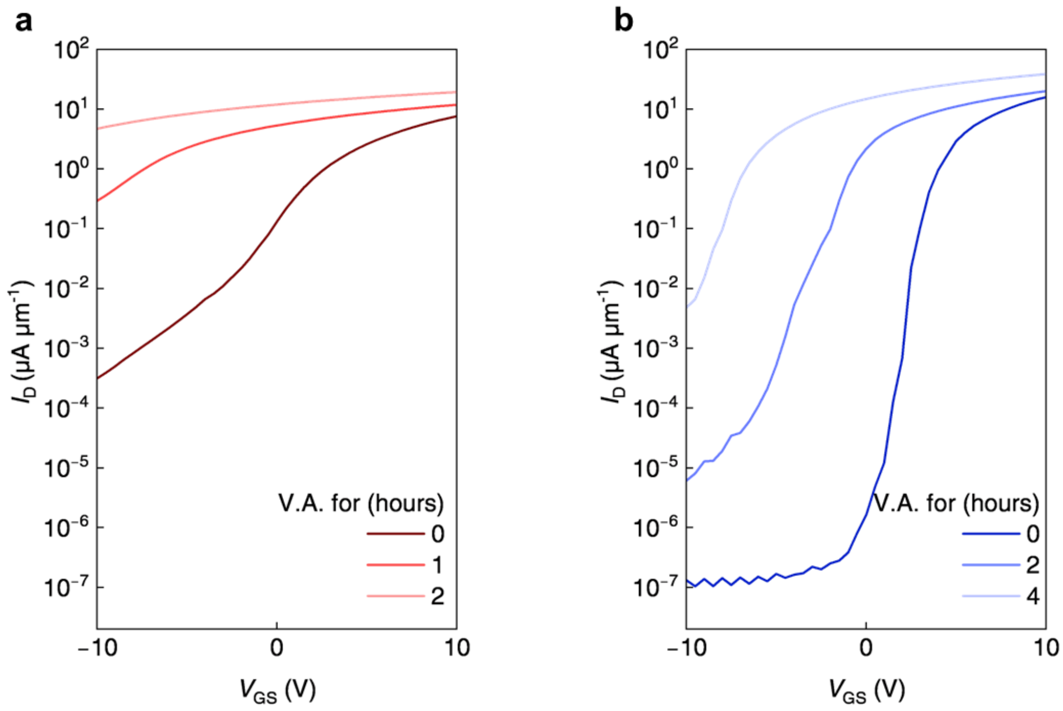

Supplementary Figure 8. a,b, Current–voltage transfer curves as a function of annealing time at 250 °C for V_{Se}-Bi_2O_2Se FET **(a)** and N-Bi_2O_2Se FET **(b)**.

The generality of this passivation strategy

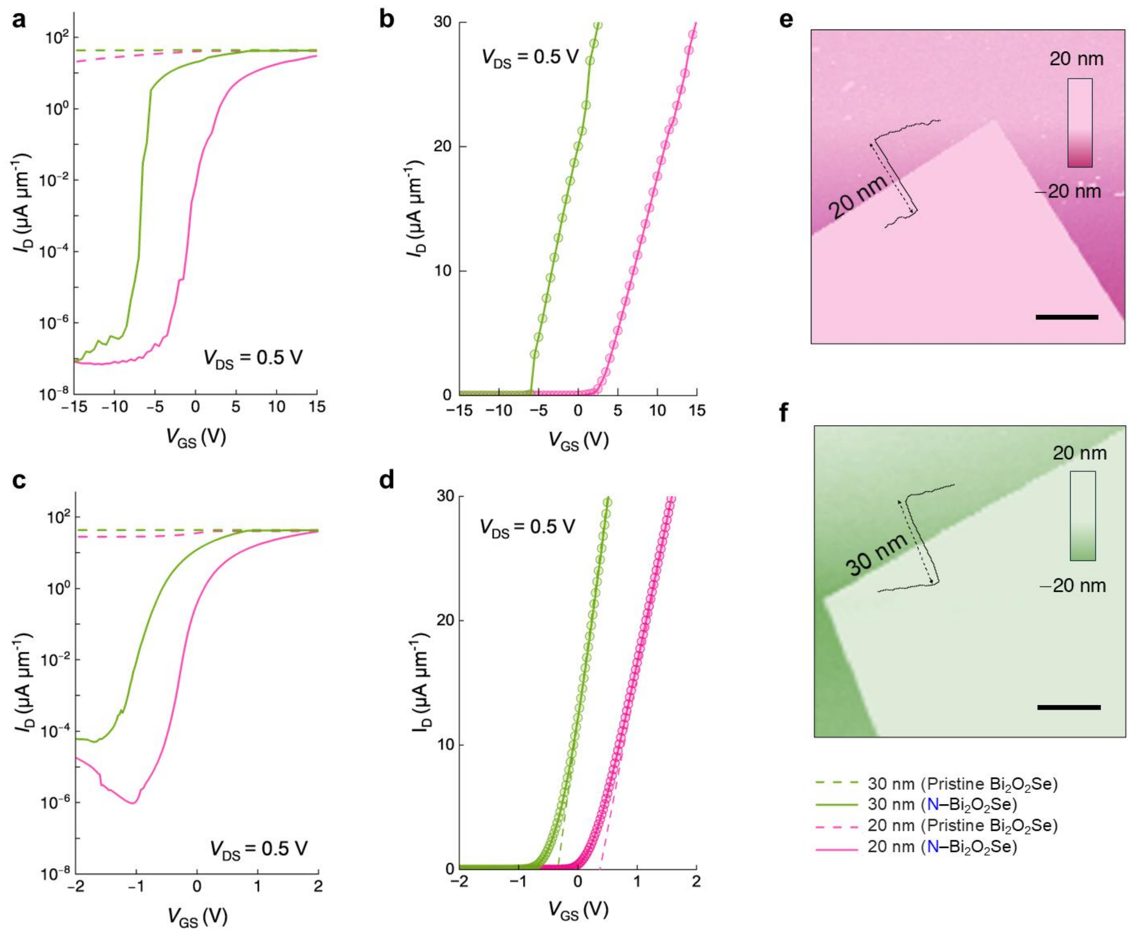

Supplementary Figure 9. a,b, Subthreshold **(a)** and transfer **(b)** (I_D–V_{GS}) curves of a V_{Se} -Bi_2O_2Se FET device with a N–Bi_2O_2Se channel integrating with 50 nm of SiO_2/p++ Si as back-gate (50 nm). **c,d,** Subthreshold **(c)** and transfer **(d)** (I_D–V_{GS}) curves of a V_{Se}-Bi_2O_2Se FET device with a N–Bi_2O_2Se channel integrating with 50 nm of HfO_x/p++ Si as back-gate (15 nm). Noted that the channel body in the devices are 30 nm and 20 nm of V_{Se}-Bi_2O_2Se, denoted as green and pink correspondingly, with the dash line representing the pristine channel. **e,f,** AFM height profile of CVD-grown Bi_2O_2Se with a thickness of 20 nm **(e)** and 30 nm **(f)**.

Monolithic 3D integration of BEOL-compatible Bi_2O_2Se on Si FinFET platforms

Supplementary Figure 10 demonstrates the fabrication process of M3D CMOS inverter, composing a Si FinFET and a local back-gate Bi_2O_2Se. A Si FinFET was first fabricated on a SiO_2/Si substrate, after which a PECVD SiO_2 film was introduced as an intermediate passivation layer and subsequently thinned and planarized by chemical–mechanical polishing. Monolithic inter-tier vias (MIVs) were then realized by etching contact holes and filling them via e-beam evaporation to form vertical interconnects. Next, a local back-gate was fabricated with a 20 nm HfO_x gate oxide, followed by transferring a Bi_2O_2Se channel onto the prepared back-gate region. Finally, the source and drain electrodes were defined on the Bi_2O_2Se using e-beam lithography and deposited by e-beam evaporation. Supplementary Figure 11 presents SEM images of the as-fabricated 3D CMOS inverter, which consists of a p-type Si FinFET positioned adjacent to a local back-gate device.

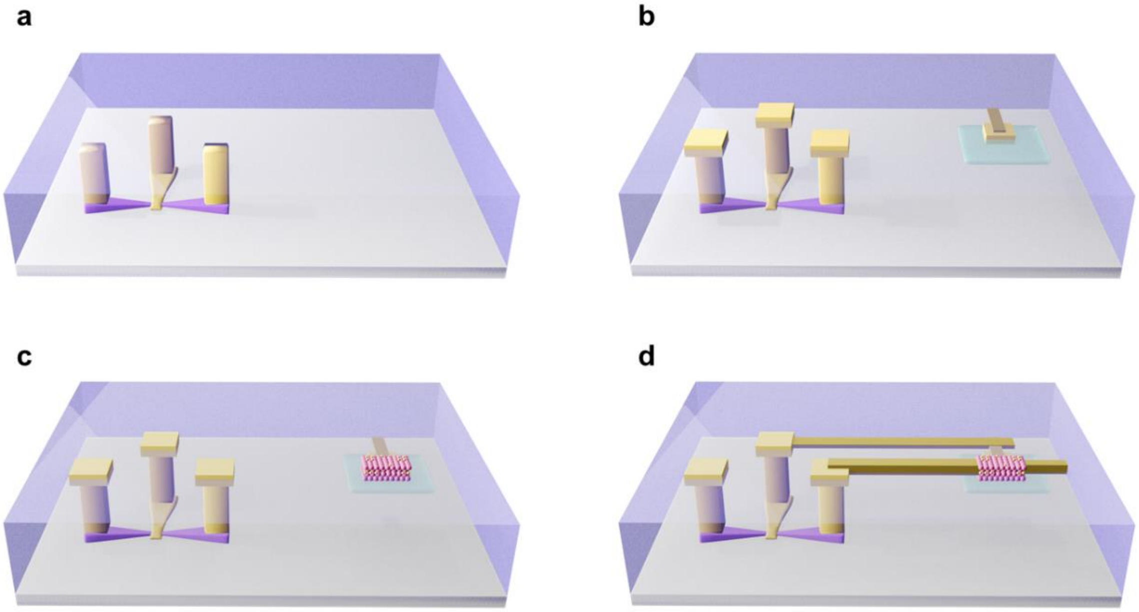

Supplementary Figure 10. Schematic and process flow of the 3D Si-Bi_2O_2Se CMOS inverter. **a,** A Si FinFET was fabricated on a SiO_2/Si substrate. An SiO_2 layer was deposited by PECVD to serve as an intermediate passivation layer, then thinned and planarized using chemical mechanical polishing. Monolithic interter vias (MIVs) were formed through contact hole etching and e-beam evaporation. **b,** Fabrication of local back-gate with the gate oxide is 20 nm of HfO_x. **c,** Transfer of a Bi_2O_2Se channel onto the as-fabricated local back-gate. **d,** Source and drain electrodes were patterned and deposited on the Bi_2O_2Se using e-beam lithography followed by e-beam evaporation.

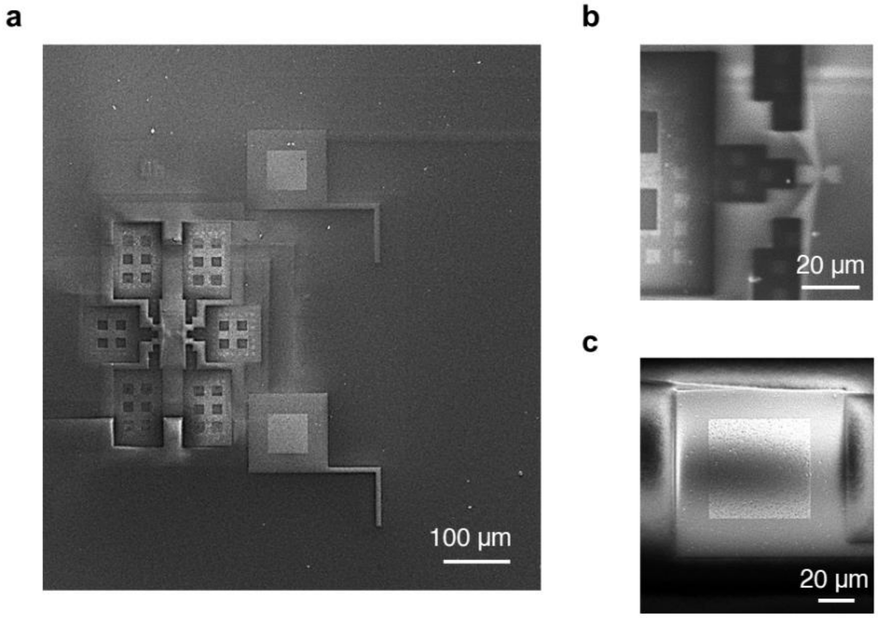

Supplementary Figure 11. a, SEM image of the fabricated 3D CMOS inverter from the top-view (scale bar: 100 μm). **b,** SEM image of the Si FinFET (scale bar: 20 μm). **b,** SEM image of the local back-gate HfO_x (scale bar: 20 μm).

Supplementary Fig. 12a depicts the circuit concept of a CMOS inverter constructed by stacking a p-type Si FinFET with a local back-gate Bi_2O_2Se transistor. In this complementary configuration, the pull-up network is provided by the PMOS and the pull-down network is provided by the NMOS, and each device can be operated in either enhancement or depletion mode depending on the biasing and device characteristics. Following positive logic conventions, a Boolean “1” is represented by V_{dd}, while a Boolean “0” corresponds to 0 V (ground). The inverter threshold voltage V_{th} is defined as the switching point where the output begins to change state, typically near $V_{dd}/2$. As summarized in Supplementary Fig. 12b, when $V_{in} < V_{th}$, the inverter output is driven high, whereas for $V_{th} < V_{in} < V_{dd}$ the output is driven low.

The operating mechanism can be understood from the conduction states of the two transistors. For a low input voltage, the nFET is intended to be in the off state and the pFET remains on, allowing current to charge the output node so that V_{out} gradually approaches V_{dd}. When the input voltage is high, the pFET turns off while the nFET turns on, enabling current to discharge the output node and pulling V_{out} down toward GND. In the intermediate input range, both transistors can conduct simultaneously, producing the sharp transition region and high voltage gain that are characteristic of an ideal CMOS inverter. In the present device stack, however, the Se-vacancy Bi_2O_2Se transistor behaves as an “always-on” channel, which weakens the pull-down/pull-up balance and leads to a markedly reduced inverter gain. Introducing nitrogen into Bi_2O_2Se helps tune the threshold voltage and restore better switching behavior, thereby increasing the gain and improving the inverter transfer characteristics (Supplementary Figure 12g-f).

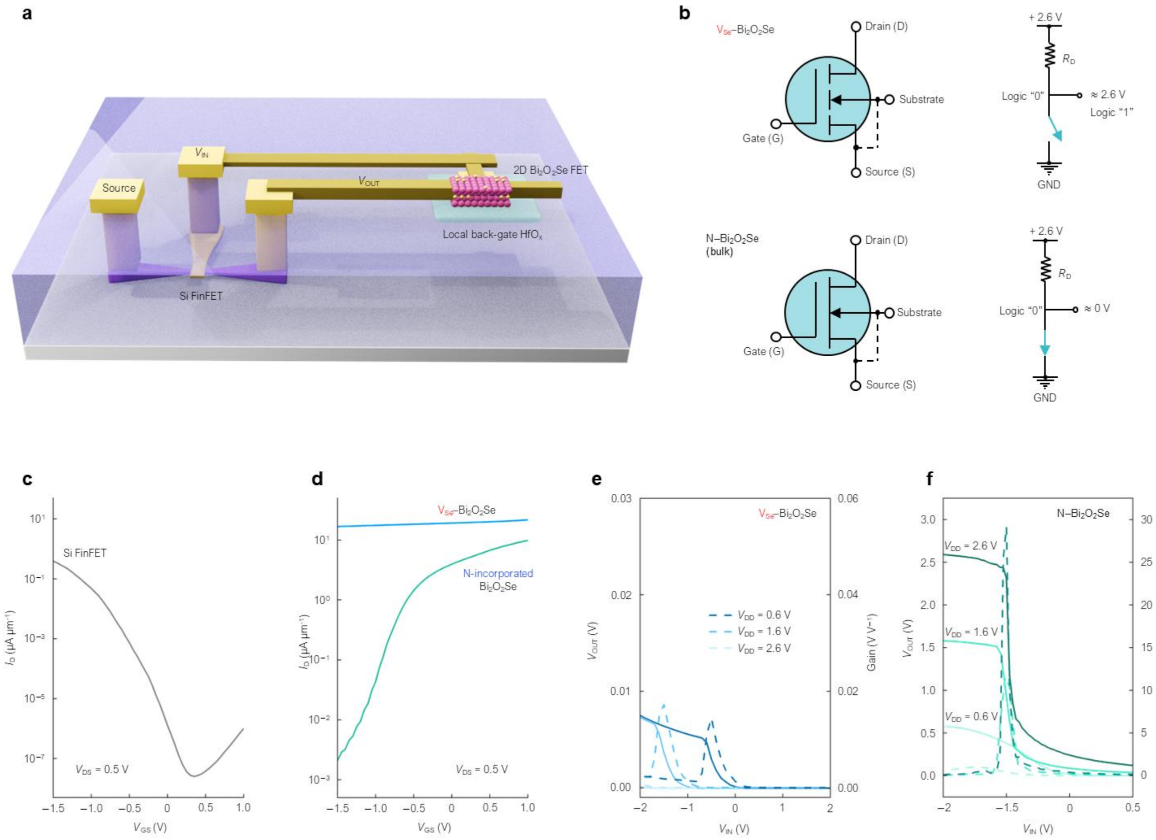

Supplementary Figure 12. Performance of Si- and 2D-Bi_2O_2Se-based 3DIC. a, Schematic of a representative CMOS inverter fabricated with a p-type Si FinFET and a n-type 2D-Bi_2O_2Se FET. **b,** Diagrams illustrating CMOS operation with varying N-channel states by modulating the electrical performance of 2D-Bi_2O_2Se FET with nitrogen adsorption. **c,d** Subthreshold transfer (I_D–V_{GS}) curves of the Si FinFET **(c)** and 2D-Bi_2O_2Se FET **(d)**. **e,f** Transfer characteristics of an inverter with the n-type 2D-Bi_2O_2Se FET before **(e)** and after nitrogen adsorption **(f)** operating at different V_{DD} varying from 0.6 to 2.6 V (left). Calculated gain ($|dV_{OUT}/dV_{IN}|$) of the inverter as a function of V_{IN} at different V_{DD} (right).

To quantify the contact transparency more rigorously, we analyzed the injection behavior using both temperature-dependent transport and transfer-length-method measurements (see Supplementary Figs 13). Arrhenius fitting yields an apparent negative barrier height, indicating that the current is not governed by thermionic emission over a positive Schottky barrier. We do not interpret this as a physical negative barrier; rather, it reflects that thermionic emission is not the dominant injection mechanism. Consistently, the devices exhibit linear low-bias I_D–V_{DS} characteristics and enhanced on-state current upon cooling. In addition, TLM extraction shows that the contact resistance scales approximately with the square root of sheet resistance, $R_C \propto \sqrt{R_{sh}}$, as expected for current spreading under transparent contacts; fitting the extracted data gives $R^2 \approx 0.99$. This scaling is consistent with current injection governed mainly by lateral current spreading in the semiconductor under the contact, rather than by a large interfacial barrier. The extracted ρ_C values remain within a narrow range, indicating that the metal/semiconductor interface remains nearly unchanged across devices. This is consistent with a contact-transparent regime, while R_C changes mainly because R_{sh} changes, rather than from a varying interfacial barrier. For the three measured sheet resistances,

$$R_{sh} = 0.5930,\ 0.2143,\ 0.1357$$

$$R_c = 2.4397,\ 1.2708,\ 1.0893$$

A linear fit of $R_c$vs $\sqrt{R_{sh}}$ gives approximately:

$$R_C \times 3.47 \approx \sqrt{R_{sh}} - 0.25$$

In the contact-transparent limit:

$$R_C \propto \sqrt{R_{sh}}$$

$$\rho_C \approx \frac{R_C^2}{R_{sh}}$$

Where:

R_C: contact resistance

p_C: specific contact resistivity

R_{sh}: sheet resistance under the contact

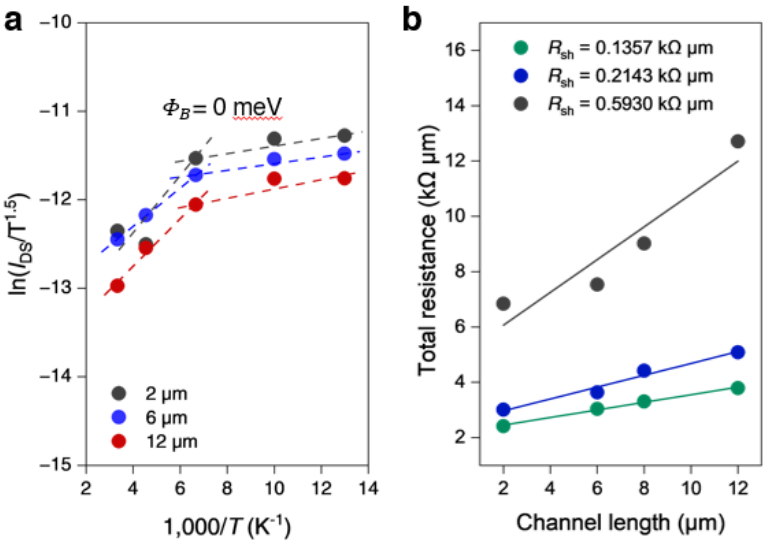

Supplementary Figure 13. a, Arrhenius plots of the ohmic N–Bi_2O_2Se FETs with V_{DS} of 0.5 V at different sheet carriers with a channel length of 2 μm (black), 6 μm (blue), and 12 μm (red). **b,** Contact resistance (R_C) extraction using the transfer-length method (TLM) for N–Bi_2O_2Se FETs on 50-nm-thick SiO_2 dielectrics. The circles are total resistance versus channel length at varied sheet resistance

Transport regimes

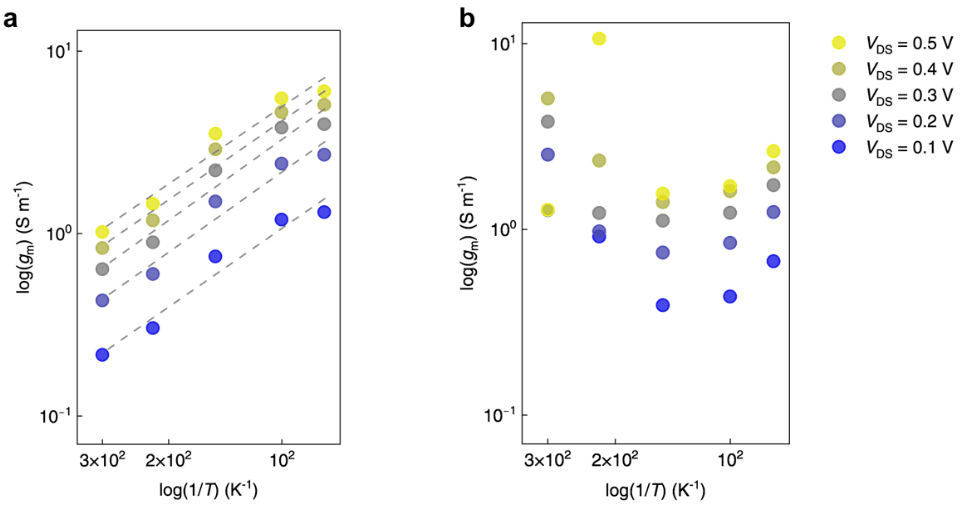

Supplementary Figure 14. a, Band-like regime: log–log plot of channel transconductance g_m vs temperature T (linear/ohmic region, constant overdrive), yields $g_m \propto T^{-\alpha}$; the fitted slope gives −1.44 (parallel traces at different V_{ds} confirm $g_m \propto V_{ds}$). **b,** Band-like ↔ Hopping regime: log–log plot of channel transconductance g_m vs temperature T, yields $g_m \propto T^{-\alpha}$; the fitted slope gives −1.44 at high-T and −0.64 at low-T, which indicates the phonon scattering domination at High-T.

Table S1. Summary of mobility, threshold voltage V_{th}, and I_{ON}/I_{OFF} of the V_{Se}-Bi_2O_2Se, N-Bi_2O_2Se (surface), and N-Bi_2O_2Se (bulk) FETs.

Sample	V_{Se}–Bi_2O_2Se	N–Bi_2O_2Se (surface)	N–Bi_2O_2Se (bulk)
**Mobility (cm^2 V^{-1} s^{-1})**	210	153	166
**V_{th} (V)**	−16.74	−5.70	9.60
**I_{ON}/I_{OFF}**	10^0	10^5	10^9

Table S2. Trade-off between mobility and I_{ON}/I_{OFF} induced by *n*- and *p*-type doping across diverse 2D semiconductors

**Channel materials**	**Dopant**	**Mobility (cm^2 V^{-1} s^{-1})**		**I_{ON}/I_{OFF}**		**Reference**
		**Before doping**	**After doping**	**Before doping**	**After doping**	
In_2O_3	O (p)	54	49	1	6	[1]
MoS_2	O (p)	13.2	14.5	5	8	[2]
MoS_2	TiOPc (p)	N/A	N/A	3	5	[3]
C_{16} IDT-BT	DMSO-HBr (n)	0.14	0.37	4	3	[4]
Cl_2–NDI	N-silane (n)	1.4	2.6	6	3	[5]
MoS_2	Y (n)	54	N/A	8	1	[6]
MoS_2	BV (n)	157.01	233.86	3	1	[7]
PCBM	PCNI2-BTI (n)	0.052	0.17	4	4	[8]
OSCs	PAD+ (n)	0.48	0.65	5	4	[9]

References

1. Tseng, R., Wang, ST., Ahmed, T. et al. Wide-range and area-selective threshold voltage tunability in ultrathin indium oxide transistors. Nat. Commun **14**, 5243 (2023).
2. Shen, PC., Lin, Y., Su, C. *et al.* Healing of donor defect states in monolayer molybdenum disulfide using oxygen-incorporated chemical vapour deposition. *Nat Electron* 5, 28–36 (2022).
3. Park, J. H. *et al.* Defect passivation of transition metal dichalcogenides via a charge transfer van der Waals interface. *Sci. Adv.* **3**, e1701661 (2017).
4. Sakai, N. *et al.* Adduct-based p-doping of organic semiconductors. *Nat. Mater.* **20**, 1248–1254 (2021).
5. He, T. *et al.* Site-specific chemical doping reveals electron atmospheres at the surfaces of organic semiconductor crystals. *Nat. Mater.* **20**, 1532–1538 (2021).
6. Jiang, J. *et al.* Yttrium-doping-induced metallization of molybdenum disulfide for ohmic contacts in two-dimensional transistors. *Nat. Electron.* **7**, 545–556 (2024).
7. Jang, J. *et al.* Reduced dopant-induced scattering in remote charge-transfer-doped MoS_2 field-effect transistors. *Sci. Adv.* **8**, eabn3181 (2022).
8. Feng, K. *et al.* Non-fullerene electron-transporting materials for high-performance and stable perovskite solar cells. *Nat. Mater.* **24**, 770–777 (2025).
9. Wang, XY., Ding, YF., Zhang, XY. *et al.* Light-triggered regionally controlled n-doping of organic semiconductors. *Nature* **642**, 599–604 (2025).
